# Microstructure and Properties of Electromagnetic Field-Assisted Laser-Clad Norem02 Iron-Based Cemented Carbide Coating

**DOI:** 10.3390/ma16206774

**Published:** 2023-10-19

**Authors:** Zixue Wang, Wanyuan Gui, Jiacheng Fu, Ping Zhu, Yonghao Lu

**Affiliations:** 1 National Center for Materials Service Safety, University of Science and Technology Beijing, Beijing 100083, China; 2 Suzhou Nuclear Power Research Institute, Suzhou 215004, China

**Keywords:** laser cladding, electromagnetic stirring, iron-based cemented carbide, microstructure, wear resistance

## Abstract

An electromagnetic field-assisted (EMF-assisted) laser cladding technique was used to prepare Norem02 iron-based cemented carbide coatings on 304 stainless steels. The coatings then were characterized in terms of their microstructure, microhardness, residual stress, and wear resistance. The results indicated that EMF did not change the phase composition of the Norem02 iron-based cemented carbide coating, but significantly affected its microstructure and properties. EMF accelerated the formation of more uniform and refined microstructure. With an increasing current intensity of EMF to 40 A, the dendritic and columnar crystal structure of the coating gradually transformed into uniform and fine equiaxed grains. However, when the EMF current intensity was increased to 80 A, a small number of small dendrites and columnar crystals began to appear at the top and bottom of the coating. Accordingly, the microhardness first increased, then decreased, and achieved a max of 376.9 HV_0.2_ at EMF current intensity of 40 A. EMF also improved the wear resistance of the coatings, reduced the cracking sensitivity, and reduced residual stress on the surface by 45.2%.

## 1. Introduction

Nuclear valves are essential parts of nuclear equipment, and their sealing surface quality directly impacts their service life. Nuclear valves often have a layer of material with improved performance melted onto their sealing surfaces in order to extend their service life [1]. Because of their highly wear- and corrosion-resistant properties, cobalt-based alloys were often used to reinforce the sealing surfaces of nuclear valves [2]. However, cobalt is a rare and expensive metal, and ^60^Co can easily be activated from ^59^Co in its wear and corrosion debris under radiation environments. ^60^Co will result in a longer half-life for radioactive nuclides, an increase in maintenance time for nuclear power plants, and a decrease in the safety of maintenance personnel [3]. Cobalt-based alloy alternatives are therefore urgently needed. It is expected that iron-based alloys will replace cobalt-based alloys, not only because of the low material price, but also the excellent wear and corrosion resistance [4,5]. At present, some studies have shown that some iron-based materials have similar properties to cobalt-based materials and could replace cobalt-based materials for engineering applications [6,7,8].

The surfacing quality and production efficiency of nuclear valve sealing surfaces are not only determined by the surfacing materials, but also by advanced surfacing processes and high-efficiency automatic surfacing equipment. A high-speed laser cladding (LC) technique has many advantages, including high efficiency, energy concentration, and small heat-affected zones, which make it an excellent surface modification tool [9]. Due to a high temperature gradient and high solidification rate [10], the resulting coatings solidification structure is prone to form directional and coarse columnar dendrites [11]. Consequently, coatings are reduced in mechanical properties, as well as more likely to develop cracks, pores, and other defects, which adversely affect the coating’s quality. In this regard, it is of significant importance to refine the solidification microstructure of iron-based alloy coatings. Previous studies focused on improving the solidification structure of laser-cladded coatings through materials design, process parameter optimization, and other techniques [12,13]. It was, however, still difficult to completely eliminate structural defects simply by changing the existing process. It is therefore necessary to consider external fields to regulate the solidification structure, thereby improving coating quality. An electromagnetic field (EMF) has the advantages of combining techniques, good controllability, and environmental friendliness, and has been used in casting, welding, and laser processing [14,15]. Using EMF-assisted laser cladding, Huang et al. [16] demonstrated that stirring could be induced on the melt pool, which might trigger the initiation of columnar dendrites, the formation of refined dendrites and equiaxed grains, as well as a more uniform distribution of temperatures. This could result in a reduction in the number of defects in the coating and a significant improvement in the wear resistance of the coating. According to previous studies, EMF assistance improved coating performance in reducing residual stress, inhibiting pores and cracks, reducing surface roughness [17], and improving corrosion and wear resistance [18,19,20,21]. Zhou et al. [22] found that the EMF-assisted laser cladding mainly contributed to electromagnetic stirring and Joule thermal effect, and an appropriate magnetic field intensity contributed to stirring the molten pool and refining the grains. The grain size of the coating tended to grow when the joule heat effect exceeded the stirring effect, resulting in a reduction in the microhardness and wear resistance of the coating as well.

In order to further study the effect of electromagnetic field on the microstructure and properties of iron-based alloy coatings, this study used EMF-assisted laser cladding technology to prepare Norem02 iron-based cemented carbide coatings on 304 stainless steels. Norem02 is an iron-based welding consumable used in nuclear power plant systems, and is widely used in domestic and foreign nuclear power plants such as Flamanville (EPR) nuclear power plant in France, units 1 and 2 of the Taishan Nuclear Power Plant, etc. So, research findings can provide reference for further optimizing the external energy field-assisted laser cladding industry and future nuclear power plant surfacing engineering.

## 2. Materials and Methods

### 2.1. Materials Preparation

**Figure 1:**
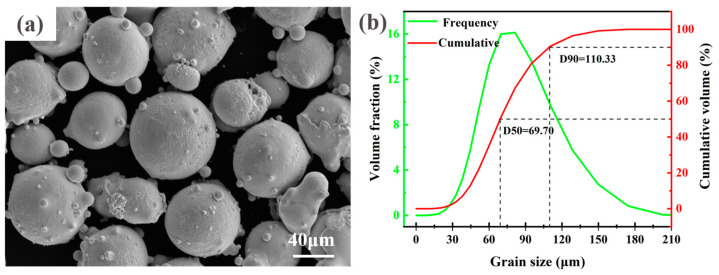
(**a**) Scanning electron microscope image and (**b**) particle size distribution of Norem02 iron-based powder.

The 304 austenitic stainless steels were used as the substrate material (10 mm ×150 mm ×200 mm). The surface was cleaned with anhydrous ethanol before the laser cladding in order to remove surface oil stains and oxides. A Norem02 iron-based alloy spherical powder with particle sizes ranging from 15 µm to 175 µm was used as the laser cladding material, as shown in Figure 1. Inductively Coupled Plasma Optical Emission Spectrometer (ICP-OES, Agilent, Beijing, China) testing was conducted on Norem02 iron-based alloy powder, and the results are shown in Table 1. The powder was dried and kept warm in a vacuum drying oven at 120 °C for four hours before the laser cladding.

### 2.2. Preparation of Norem02 Iron-Based Cemented Carbide Coating

**Figure 2:**
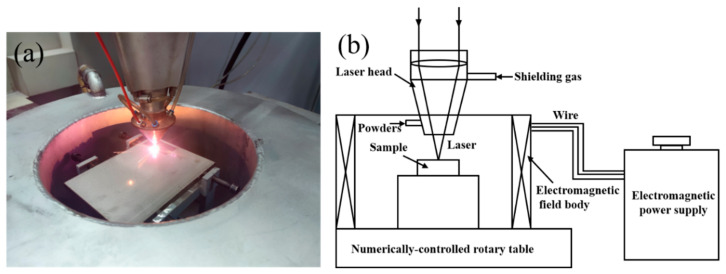
(**a**) The high-speed laser cladding complete system and (**b**) schematic diagram of EMF-assisted laser cladding scheme.

All experimental samples were prepared utilizing a high-speed laser cladding system (ZKZM-4000, ZKZM, Xi’an, China). A computer numerical control (CNC) machine tool controlled the movement of the cladding head, and Norem02 iron-based powder was fed into the molten pool via a powder feeder synchronized with the movement of the cladding head. The cladding process was carried out in an argon atmosphere. The laser process parameters were fixed to analyze the differences in microstructure of the Norem02 iron-based cemented carbide coating before and after the application of EMF, and the influence of current intensity on the microstructure evolution of the coating. A specific process was employed at a laser power of 1600 W, a scanning rate of 0.6 m/min, a spot diameter of 2 mm, a powder feeding rate of 5.1 g/min, and an overlap rate of 50%. Molten pools were protected by argon gas protection (purity 99.99%) with a flow rate of 12.5 L/min. Figure 2 illustrates a specially designed laser cladding auxiliary magnetic field generation device, which changes the intensities of the current of 0 A, 40 A, and 80 A to obtain the desired magnetic field size. The specific process parameters are shown in Table 2.

### 2.3. Characterizations

After laser cladding, the forming part was cut into 10 mm × 10 mm × 10 mm with an electric spark wire cutting machine. The specimen was first ground and polished, and then corroded in saturated oxalic acid solution (constant voltage 5 V) for 10 s to 20 s to observe the microstructure. The microstructure changes were observed using an optical microscope (OM) (Olympus Corporation BX53M, Tokyo, Japan) and a scanning electron microscope (SEM) (with energy dispersive spectroscopy (EDS) (Zeiss Merlin Compact, Oberkochen, Germany). In order to determine the phase composition of the coatings, X-ray diffraction (XRD, Rigaku Smart Lab, Tokyo, Japan. 20°/min step angle; 2θ = 20° to 100° scanning range) was used.

A Qpix hardness tester was used for microhardness testing, one test point was taken every 200 µm from the coating surface to the substrate, and every point was tested three times. The load was 200 g, and the loading time was 15 s. An X-ray diffraction method was used to detect residual stresses on the surface of the coating using a residual stress detector (iXRD, supplied by Proto Manufacturing, Lasalle, ON, Canada). It must be noted that normal sample handling results in the release of stress, which results in inaccurate residual stress values. Therefore, residual stress was tested before mechanical cutting was performed.

Friction and wear tests were conducted on a Rtec friction and wear testing machine. Before the test, the surface of the cladding layer was polished to a mirror finish. Friction and wear tests were conducted at room temperature without lubrication. A ZrO_2_ ceramic ball was used as the grinding material, the load was 25 N, the frequency was 5 Hz, the friction distance was 4 mm, and the wear time was 20 min. After the friction and wear tests, the samples were soaked in alcohol, underwent ultrasonic cleaning for 10 min, and the microstructure of the coating surface was analyzed by OM. The wear volume was calculated using a laser confocal scanning microscope (LSCM, KEYENCE, Shanghai, China) The wear surface and cross-section were observed by scanning electron microscope (SEM) (Zeiss Merlin Compact, Oberkochen, Germany). The element composition was analyzed by energy dispersive spectrometer (EDS) (Zeiss Merlin Compact, Oberkochen, Germany).

## 3. Results and Discussion

### 3.1. Microstructure

**Figure 3:**
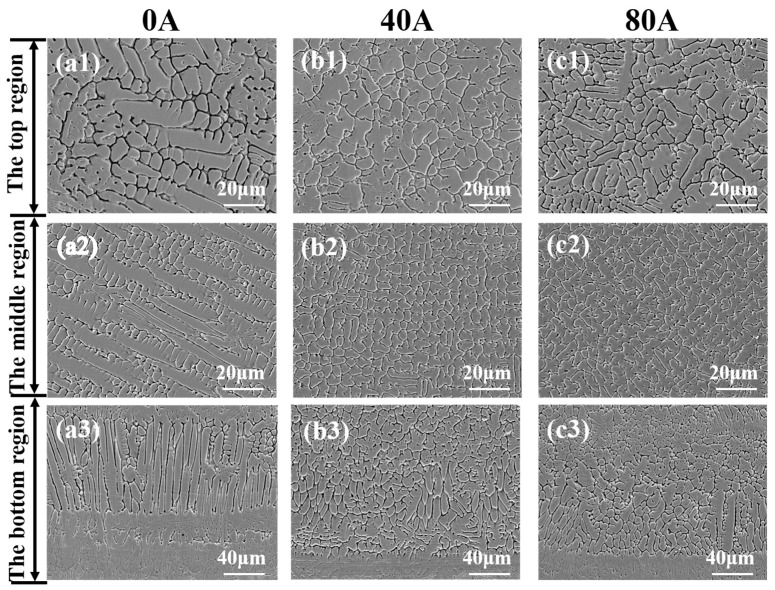
The cross-sectional SEM morphologies in the top, middle, and bottom of the coatings with different EMF current intensities: (**a1**–**a3**) I = 0 A; (**b1**–**b3**) I = 40 A; (**c1**–**c3**) I = 80 A.

Figure 3 shows that laser cladding the Norem02 coating displays different microstructures under various magnetic field intensities. Without the assistance of EMF, as shown in Figure 3(a1–a3), the top of the coating is composed of coarse equiaxed grain and bits of dendrites. A large number of fishbone-like columnar dendrites are produced in the middle. The bottom structure is composed of columnar crystals growing perpendicular to the interface. It exhibits typical characteristics of laser cladding solidification structures. Different regions of the laser cladding coating have a different microstructure depending on their temperature gradient to solidification rate ratio (G/R) [23]. When the EMF current is 40 A, as shown in Figure 3(b1–b3), the coarse equiaxed grain and dendrites at the top of the coating are gradually refined to fine equiaxed grain. A larger number of grain refinement areas appears in the central columnar dendrite. The columnar crystals at the bottom of the coating are significantly refined to a large number of cellular crystals perpendicular to the interface. When the EMF current is 80 A, as shown in Figure 3(c1–c3), a small number of small dendrites begin to appear at the top of the cladding layer. The small columnar crystals begin to sprout at the bottom, accompanied by a trend in grain growth.

When the current of the EMF is 40 A, the grain refinement and uniform microstructure of the cladding layer are achieved, because the external EMF changes the distribution of the temperature field in the cladding layer. This occurs by affecting the mass transfer, heat transfer, and flow of the molten pool, which enhances material exchange within the molten pool to a certain extent and promotes the uniform cooling and solidification of the cladding layer. According to the theory of grain nucleation [23,24] and the Hartmann effect of a magnetic-controlled melting pool [25], increasing the intensity of magnetic field leads to an increase in liquid phase convection near the surface of the molten pool. The erosion effect of liquid metal on the crystallization and the mechanical damage degree of cylindrical dendrite cells is enhanced, which leads to the increase in equiaxed crystal and the grain refinement. When the EMF current is 80 A, as shown in Figure 3(c1–c3), the coating begins to show a trend of grain growth due to the increase in the induced current in the laser molten pool induced by the increase in magnetic field intensity, resulting in a Joule heat effect during the solidification process and decreasing the subcooling of the liquid Norem02 iron-based cemented carbide in the laser molten pool. The reduced subcooling within the laser melt pool will provide favorable conditions for the growth in broken or shattered crystal blocks, resulting in a grain growth trend in the cladding layer [26].

**Figure 4:**
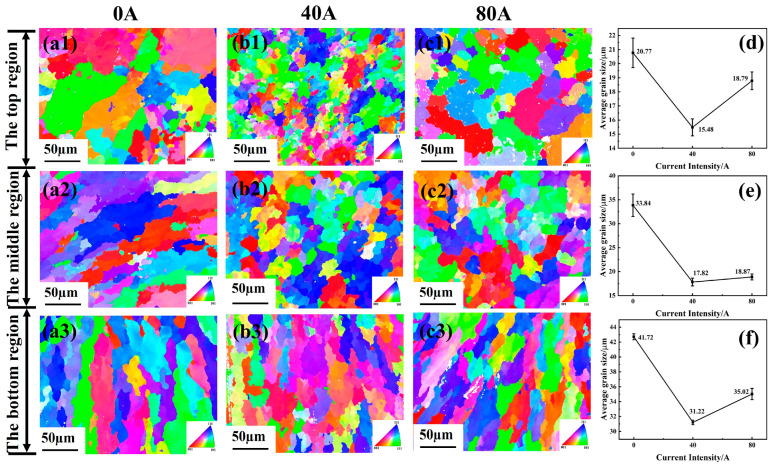
The cross sectional IPF maps and average grain size (**d**–**f**) in the top, middle, and bottom of coatings with different EMF current intensities: (**a1**–**a3**) I = 0 A; (**b1**–**b3**) I = 40 A; (**c1**–**c3**) I = 80 A.

Figure 4 shows that the IPF map and average grain size of the top, middle, and bottom of coatings with different EMF current intensities. It is found that, with the assistance of EMF, the microstructure at the top and middle of the coatings becomes more uniform and refined, and the size of the elongated columnar crystals at the bottom is also significantly reduced. However, when the EMF current is 80 A, the grains in each area of the coating show a slight growth compared with the coating with an EMF current intensity of 40 A, which is consistent with the results in Figure 3. The average grain sizes of the top, middle, and bottom of coatings with different EMF current intensities are shown in Figure 4d–f, respectively. The results indicate that the grain size of each area shows a trend of first decreasing and then increasing with the increase in EMF current intensity. It is obvious EMF can homogenize and refine the microstructure grains of the coating.

**Figure 5:**
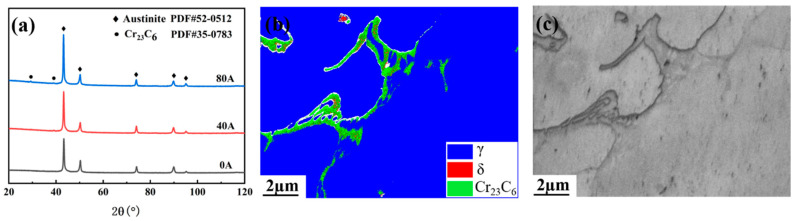
(**a**) XRD patterns of coatings with different EMF current intensities; (**b**) EBSD phase diagram, and (**c**) SEM micrographs of the coating with an EMF current intensity of 40 A.

Figure 5 shows that the comparison of the XRD patterns of laser cladding Norem02 coatings under different EMF intensities, EBSD phase diagram and SEM micrograph of the coating with an EMF current intensity of 40 A. EBSD phase diagram and SEM micrograph (Figure 5a,b) indicate that coatings are mainly composed of austinite, ferrite, and carbide (Cr_23_C_6_). The carbide (Cr_23_C_6_) mainly has a network morphology in austenite crystal, and a small amount of ferrite exists in carbide, forming a mixed zone of ferrite and carbide. Due to the low content of ferrite, only the characteristic peaks of austenite and carbide (Cr_23_C_6_) are detected by XRD. The result of XRD indicates that the EMF application during the laser cladding process has little effect on the phase composition of the coating during the solidification process of the laser melt pool. On the other hand, the diffraction peak intensities of the cladding coatings assisted by external EMF increase at approximately 43° and 97° when EMF is applied, which indicates that the EMF increases the content of solid solution [27].

### 3.2. Microhardness and Residual Stress

**Figure 6:**
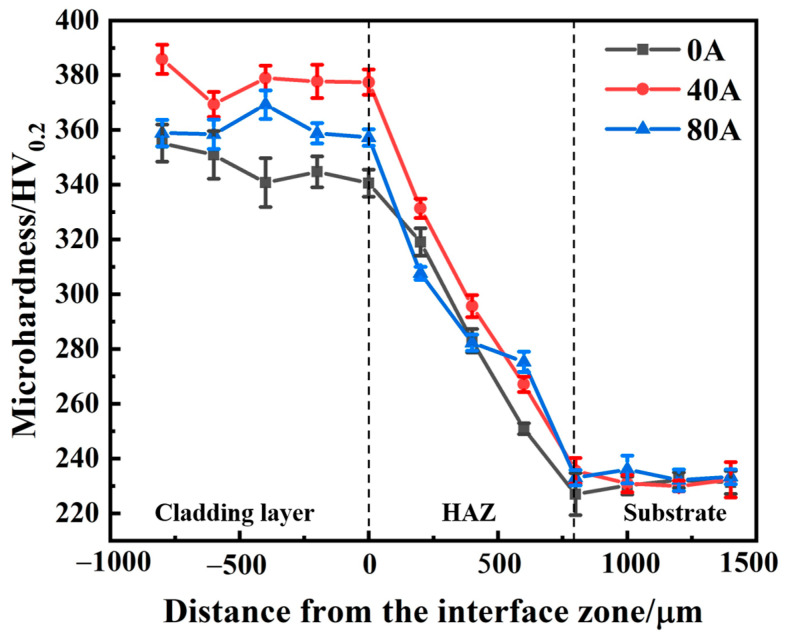
Cross sectional microhardness curves of Norem02 iron-based cemented carbide coatings with different EMF current intensities.

Figure 6 shows that the hardness distribution profile across from the coating to the heat-affected zone (HAZ) and then to the substrate. It is found that a higher hardness is obtained in the coatings compared to that of the substrate, and the hardness gradually decreases from the coating to substrate. Wherein, the average microhardness of the coating without magnetic field assistance is 346.0 HV_0.2_, and the microhardness slightly decreases from the coating surface to interface, which results from a high cooling rate, high temperature gradient, and rapid solidification of the laser cladding to form a planar crystal region, a columnar dendrite region, and an equiaxial crystal region within the Norem02 iron-based cemented carbide coating [28]. When an EMF current intensity of 40 A, the average microhardness increases to 376.9 HV_0.2_. However, when the EMF current intensity increases to 80 A, the average microhardness of the coating decreases to 360.5 HV_0.2_. Obviously, when the EMF current intensity increases, the microhardness of the coating first increases, then decreases. This is due to the formation of a more uniform microstructure induced by the increased flow and unified temperature field of the molten pool under the stir of the electromagnetic force. However, when the EMF intensity reaches 80 A, the coating hardness decreases, because the Joule heat action is greater than the EMF stirring action, which makes the grain coarse at the surface [22].

**Figure 7:**
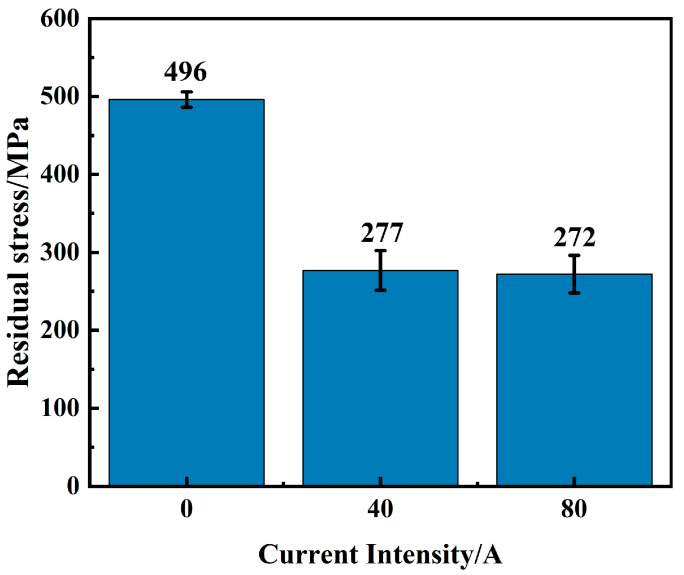
Residual stresses on surfaces of coatings with different EMF current intensities.

Figure 7 illustrates the residual stresses on the surface of the coatings under various EMF current intensities. It is found that the residual stress on the coating surfaces is tensile stress. Without EMF assistance, the residual stress on the coating surface is 496 MPa. When an EMF of 40 A is applied, residual stress on the coating surface decreases to 277 MPa, which decreases about 44.2%. With an increasing EMF current intensity to 80 A, residual stress on the coating surface slightly decreases to 272 MPa. It is found that the residual stress occurs due to rapid heating and cooling during laser cladding, and thermal effects are the main cause of residual stress [29]. Under the mechanical stirring effect of EMFs on molten pools, EMF can promote the mass transfer process within the molten pools and reduce the temperature gradient, which ensures that the distribution of solute elements and heat within the molten pool is uniform, thus reducing residual stresses.

### 3.3. Wear Performance

**Figure 8:**
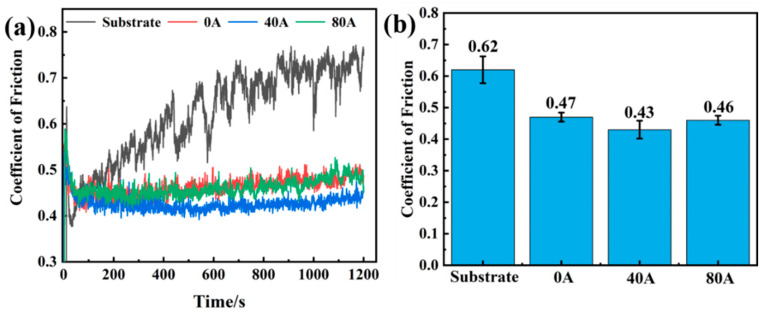
(**a**) The COF wear–time relationship and (**b**) average COF of the 304 substrate and the coatings with different EMF intensities.

Figure 8 shows the coefficient of friction (COF) wear–time relationship of the 304 substrate and the coatings with different EMF intensities. The friction and wear process can be divided into two stages: the running-in wear period and the stable wear period. Compared with that of the substrate, the COF of the coatings has been significantly reduced and changes indistinctively over time, as shown in Figure 8a. On the other hand, when EMF is applied, the COF of Norem02 iron-based cemented carbide coatings slightly decreases. Wherein, the COF of the coating is the lowest, at 0.43, when the EMF current reaches 40 A, as shown in Figure 8b.

**Figure 9:**
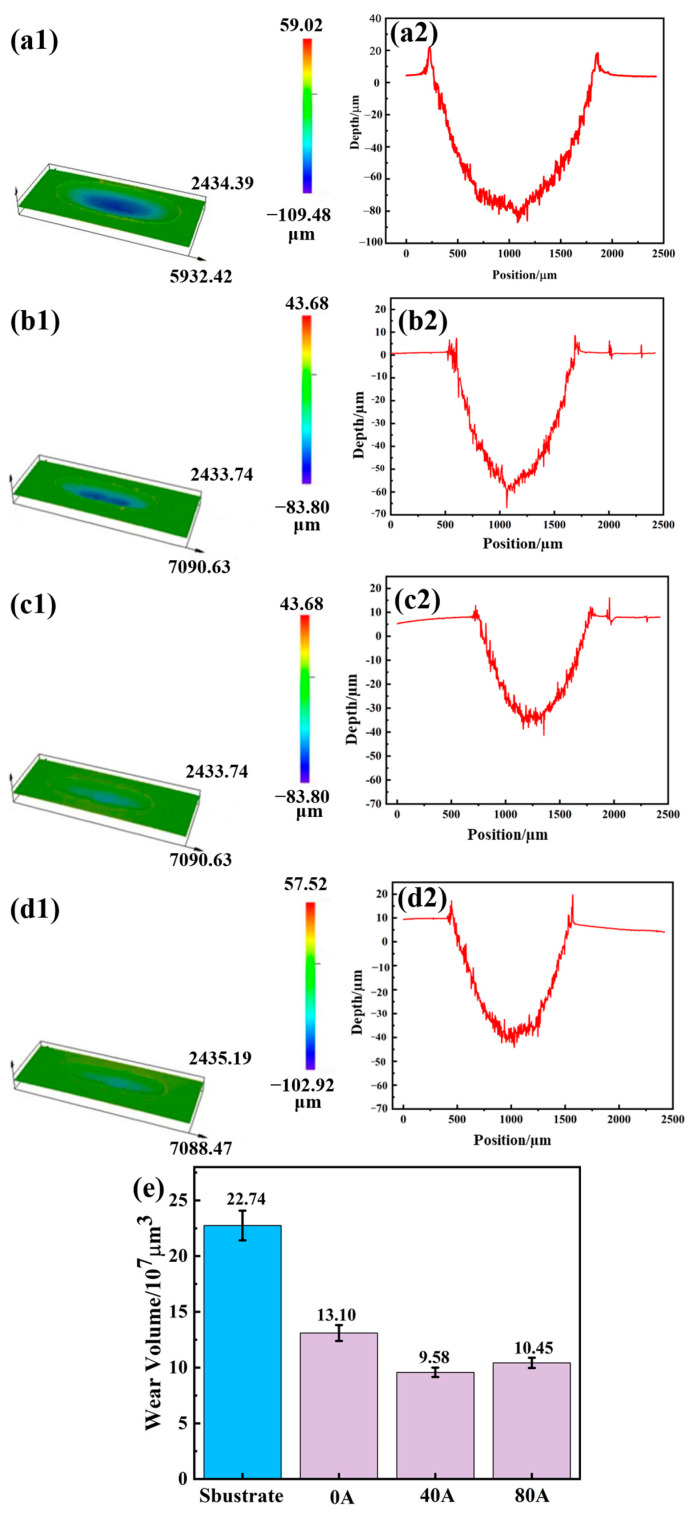
Wear 3D morphologies (**a1**–**d1**), wear depths (**a2**–**d2**) and wear volumes (**e**) of substrate and coatings with different EMF current intensities.

Figure 9 illustrates the wear morphologies and wear scar volumes of the substrate and coatings with different EMF current intensities. Both the wear depth and wear volume of the coatings are significantly lower than those of substrate and coating without EMF assistance, which indicates that EMF makes the coatings more anti-wear performance. Without EMF assistance, the wear depth of the coating is about 60 µm and the wear volume is 13.1 × 10^7^ µm^3^. When the EMF current of 40 A is applied, the wear depth of the coating decreases to about 43 µm and the wear volume decreases to 9.58 × 10^7^ µm^3^. However, when the EMF current increases to 80 A, both the wear depth and wear volume of the coating slightly increase to about 50 µm and 10.45 × 10^7^ µm^3^, respectively. According to the results, the wear resistance of the coating can be significantly enhanced by applying the appropriate EMF current intensity during the laser cladding process.

**Figure 10:**
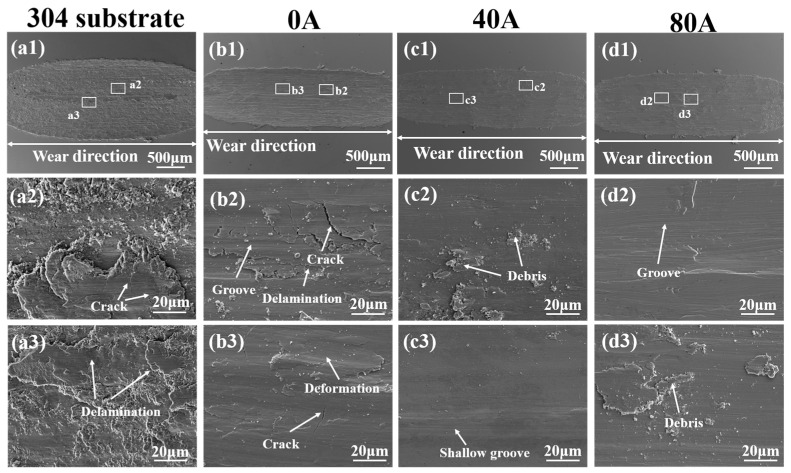
(**a1**–**a3**) Overall scar SEM wear morphologies and the magnification ones of the corresponding areas in the 304 substrate and the coatings with different EMF current intensities: (**b1**–**b3**) I = 0 A; (**c1**–**c3**) I = 40 A; (**d1**–**d3**) I = 80 A.

Figure 10 shows the overall SEM morphologies of wear scar in the substrate and coatings with different EMF current intensities and the magnification images of the worn surface. From Figure 10(a1–d1) it is clear that the degree of depression of the substrate is deepest, and the coating without EMF assistance is deeper than that of coatings with EMF assistance. As illustrated in Figure 10(a2,a3), for the 304 substrate, the delamination damage and small cracks on the worn surface indicated that the main wear mechanism is fatigue wear existing in the form of delamination. As illustrated in Figure 10(b2,b3), the coating without EMF assistance exhibits delamination and plastic deformation. Additionally, a large number of cracks perpendicular to the wear direction are found in the wear surface, indicating adhesive wear takes place. Additionally, several furrows with different depths are shown in the wear surface, which indicates that abrasive wear also occurs on the coating without EMF assistance. For the coating with an EMF current of 40 A, as shown in Figure 10(c2,c3), the wear surface appears relatively smooth, with slight furrows, and with a small amount of worn debris, and there are no visible cracks in the wear surface, which indicates that wear mechanism of the coating is mainly abrasive wear, accompanied by slight adhesive wear. In the case, the wear resistance is substantially improved. This is attributed to the mechanical stirring effect of the electromagnetic force generated by the EMF on the molten pool, which can improve the nucleation rate, uniform the temperature field of the molten pool, make the structure more uniform and refined, and contribute to fine crystal strengthening, improving wear resistance and toughness of the coating as well as preventing crack formation and expansion [30]. When the EMF current increases to 80 A, as shown in Figure 10(d2,d3), a large number of furrows and a small amount of wear debris are found in its wear surface, but there are no cracks, indicating that abrasion is the primary wear mechanism in this condition. It is obvious that the furrows in the worn surface of coating with an EMF current of 80 A are deeper than those in coating with an EMF current of 40 A, and the size of the worn debris is also larger. The reason is the coarsening of the grains at the top of coating as well as the decrease in the hardness of the coating when the EMF current increases to 80 A.

**Figure 11:**
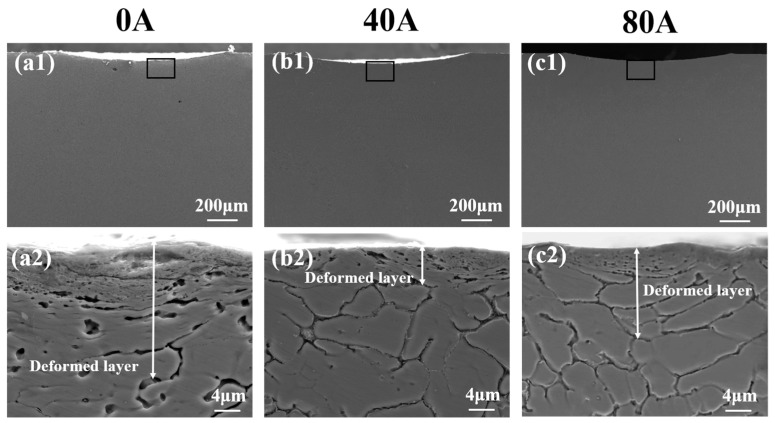
The cross-sectional SEM morphologies of wear scar of the coatings with different EMF current intensities: (**a1**,**a2**) I = 0 A; (**b1**,**b2**) I = 40 A; (**c1**,**c2**) I = 80 A.

Figure 11 illustrates cross-sectional the SEM morphologies of coatings with different EMF current intensities and magnification images of them. As shown in Figure 11(a1,a2), a thick worn debris with a thickness of about 5 µm and plastic deformation are visible underneath the wear scar in the coating without EMF assistance. The grains in the deformed layer are elongated along the direction of fretting. Several microcracks are also visible in the worn debris, as shown in Figure 11(a2). Compared with those in Figure 11(a1,a2), both the worn debris and the deformation layer thickness in Figure 11(b1,b2) of coating B are small. According to Figure 11(b2), the deformation layer of coating B is small, and there are no cracks, which indicates that its deformation resistance and wear resistance are significantly improved. However, it can be observed in Figure 11(c1,c2) that the wear debris and deformed layer of coating C become thick again, and its thickness reaches approximately 10 µm, which indicates its deformation resistance and wear resistance is lower than that of coating B and the grains in the deformed layer are elongated into thin strips.

**Figure 12:**
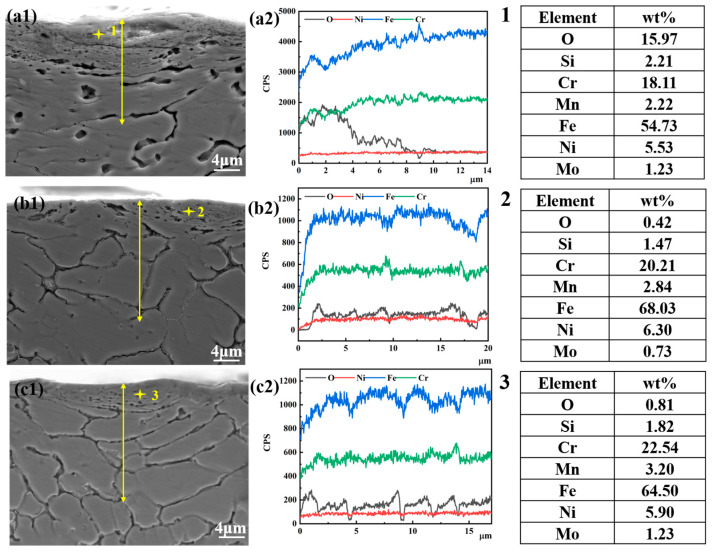
The cross-sectional element analyses of wear scar in the coatings with different EMF current intensities: (**a1**,**a2**) I = 0 A; (**b1**,**b2**) I = 40 A; (**c1**,**c2**) I = 80 A.

Figure 12 illustrates the cross-sectional distribution of elements of the wear scar of coatings with different EMF current intensities. It is found that there is a high oxygen concentration in the wear debris of the coating without EMF assistance. Comparatively, there only is a small amount of oxygen concentration in the wear cross-section of the coatings with EMF assistance, indicating that they are less oxidized to form oxides.

**Figure 13:**
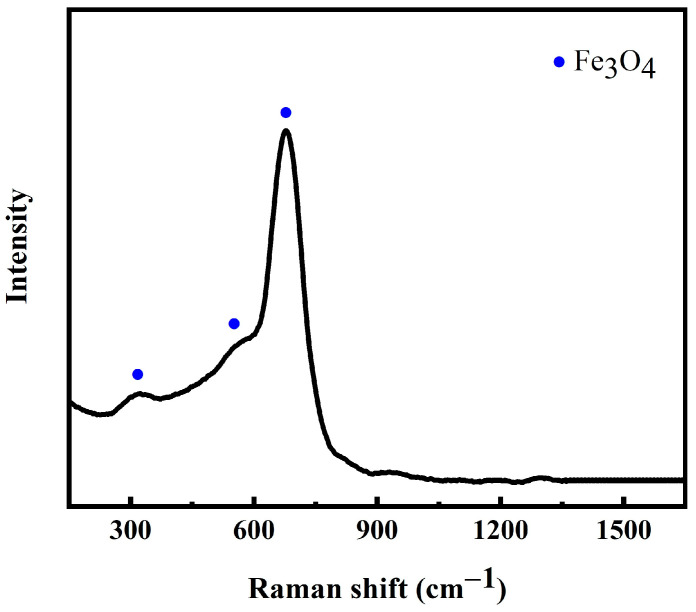
Raman mapping analyses for the oxides in cross-section wear scar of coating without EMF assistance.

Figure 13 shows the Raman mapping analyses for the oxides in cross-section of the wear scar of the coating without EMF assistance. It can be seen that only one type of oxide Fe_3_O_4_ was detected, with one major peak at the wavenumber of 677.0 cm^−1^ and two minor peaks at the wavenumber of 322.9 cm^−1^ and 547.2 cm^−1^.

## 4. Conclusions

Laser cladding technology assisted by EMF was used to prepare Norem02 iron-based cemented carbide coatings on 304 stainless steels. An investigation was conducted into the relationship between EMF and coating microstructure and properties. Conclusions can be drawn as follows:

(1) EMF did not change the phase composition of the Norem02 iron-based cemented carbide coating, but obviously changed the microstructure of coatings. When the EMF current intensity was increased to 40 A, the dendritic and columnar crystal structure of the coating gradually transformed into fine equiaxed grains, and the microstructure became more uniform and refined. However, when the EMF current intensity was increased to 80 A, a small number of small dendrites and columnar crystals began to appear at the top and bottom of the coating;
(2) The auxiliary effect of the EMF improved the microhardness of the coatings and reduced the residual stress. The microhardness of the coatings first increased, then decreased; the EMF current intensity increased and achieved the maximum at current intensity of 40 A. The residual stress on the surface of the coating decreased with the EMF current intensity increased. When the magnetic field current intensity reached 40 A, the residual stress decreased from 496 MPa to 277 MPa, which decreased by 44.2%;
(3) EMF improved wear resistance of the coatings, decreased cracking sensitivity, and changed the main wear mechanism from adhesive wear to abrasive wear. With the increase in EMF intensity, the loss amount and wear coefficient of the coating decreased first, then increased. When the EMF current reached 40 A, the wear resistance of the coating was the best, and the wear volume was 9.58 × 10^7^ µm^3^.

## Figures and Tables

**Table 1:** Chemical composition of Norem02 iron-based alloy powder.

Sample	Ni	Fe	Co	Cr	C	B	Si	Mn	Mo	N
Norem02	3.7–4.4	Bal.	≤0.05	23.0–26.0	1.10–1.13	≤0.02	3.1–3.5	4.0–5.0	1.8–2.2	0.14–0.18
ICP-OES	4.07	60.36	0	25.4		0.0097	3.3	5.13	1.84	

**Table 2:** Multiple variations in current intensity during the EMF-assisted laser cladding process.

Sample	Laser Cladding Power (W)	Laser Cladding Speed (m/min)	Powder Feeding Speed (g/min)	Overlap Ratio	Current Intensity (A)
A	1600	0.6	5.1	50%	0
B	1600	0.6	5.1	50%	40
C	1600	0.6	5.1	50%	80

## Data Availability

The data that support the findings of this study are available from the first corresponding author upon reasonable request.
